# Improvement of ultra-small-angle XPCS with the Extremely Brilliant Source

**DOI:** 10.1107/S1600577523008627

**Published:** 2024-01-01

**Authors:** William Chèvremont, Thomas Zinn, Theyencheri Narayanan

**Affiliations:** a ESRF – The European Synchrotron, 71 Avenue des Martyrs, 38043 Grenoble, France; NSRRC, Taiwan

**Keywords:** XPCS, coherent X-ray scattering, speckle contrast, UA-XPCS, USAXS

## Abstract

Recent technical developments and the performance of the X-ray photon correlation spectroscopy (XPCS) method over the ultra-small-angle range with the Extremely Brilliant Source (EBS) at the ESRF are described. With higher monochromatic coherent photon flux (∼10^12^ photons s^−1^) provided by the EBS and the availability of a fast pixel array detector (EIGER 500K detector operating at 23000 frames s^−1^), XPCS has become more competitive for probing faster dynamics in relatively dilute suspensions. One of the goals of the present development is to increase the user-friendliness of the method. This is achieved by means of a Python-based graphical user interface that enables online visualization and analysis of the processed data. The improved performance of XPCS on the Time-Resolved Ultra-Small-Angle X-ray Scattering instrument (ID02 beamline) is demonstrated using dilute model colloidal suspensions in several different applications.

## Abstract 2

The technical performance of ultra-small-angle X-ray photon correlation spectroscopy with the ESRF Extremely Brilliant Source is presented. The brighter X-ray source together with advanced pixel array detectors enable investigations of faster dynamics in relatively dilute suspensions by this method.

## 1. Introduction

X-ray photon correlation spectroscopy (XPCS) is a powerful technique for probing the equilibrium dynamics from the nanometric to micrometric scales in condensed matter systems (Narayanan & Konovalov, 2020 ▸; Lehmkühler *et al.*, 2021 ▸). Technically, it is an alternative to dynamic light scattering for the investigation of complex fluids, providing access to their wavevector-dependent dynamics (Sutton, 2008 ▸). Nevertheless, applications of XPCS have been primarily restricted to relatively slow dynamics due to limited coherent photon flux and the frame rate of high resolution X-ray detectors (Grübel *et al.*, 2008 ▸). The advent of the fourth-generation synchrotron sources (Eriksson *et al.*, 2014 ▸; Raimondi *et al.*, 2021 ▸; Liu *et al.*, 2022 ▸), together with fast pixel array detectors (Dinapoli *et al.*, 2011 ▸; Zhang *et al.*, 2018 ▸; Zinn *et al.*, 2018 ▸; Nakaye *et al.*, 2021 ▸), has overcome these limitations to some extent. As a result, XPCS can now probe relatively fast dynamics in dilute systems with sufficient scattering contrast (Zinn *et al.*, 2022 ▸) or concentrated systems with low scattering contrast (Chushkin *et al.*, 2022 ▸).

This article presents the recent technical developments related to the exploitation of XPCS using the ESRF Extremely Brilliant Source (EBS) (Raimondi *et al.*, 2021 ▸) on the Time-Resolved Ultra-Small-Angle Scattering (TRUSAXS) instrument (ID02 beamline) (Narayanan *et al.*, 2022 ▸). With the EBS, a monochromatic coherent flux of 10^12^ photons s^−1^ and a speckle contrast close to 45% in the ultra-small-angle range can be obtained. The EIGER 500K detector available at the instrument enables acquisition of up to 23000 frames s^−1^ (Zinn *et al.*, 2018 ▸). As a result, the instrument offers a somewhat unique size and time scales for XPCS investigations. Fast processing of XPCS data is facilitated by the *Dynamix* Python software package (Paleo *et al.*, 2021 ▸). Additionally, a Python software named *XPCSUtilities* (Chèvremont, 2023*a* ▸) was developed as a user-friendly graphical interface to access, visualize and analyze the processed data.

The performance of XPCS at the TRUSAXS instrument is demonstrated using dilute colloidal suspensions and powder samples. The speckle contrast is characterized as a function of sample-to-detector distance at the standard working energy of 12.230 keV, and as a function of energy at the maximum sample-to-detector distance (31 m). The results are compared with theoretical estimates (Sandy *et al.*, 1999 ▸). The stability of the beamline, and thus its ability to perform long-time XPCS, has been characterized using a fixed alumina powder sample. Furthermore, as an example of faster out-of-equilibrium dynamics, the relaxation of a stirred colloidal suspension upon shear cessation was probed. Finally, the feasibility of high-pressure XPCS from colloidal suspensions is demonstrated.

## 2. Technical background

This section describes the beamline instrumentation and software interface for performing ultra-small-angle (UA) XPCS experiments at the TRUSAXS instrument.

### 2.1. Source and optics

The ESRF-EBS is a fourth-generation synchrotron, based on the hybrid multibend achromat lattice (Raimondi *et al.*, 2021 ▸). Compared with the previous third-generation (3rd gen) source, the brightness and transverse coherence of the beam have been increased by more than an order of magnitude (Raimondi *et al.*, 2021 ▸). The enhanced brightness and coherence are very beneficial for XPCS, as this technique ideally requires a single coherence area (Berne & Pecora, 2000 ▸). The storage ring operates at an electron energy of 6 GeV with an electron beam of nominal full width at half-maximum (FWHM) emittances of about 24 pm rad and 310 pm rad in the vertical and horizontal directions, respectively (Raimondi *et al.*, 2021 ▸).

The X-ray beam at the TRUSAXS instrument is delivered by two phased undulators with a magnetic period of 21.4 mm (U21.4) and minimum gap 11 mm, which are primarily used for fixed-energy operation at 12.230 keV and up to 15 keV. The phasing of the two undulators increases the flux more than the two devices separately, and keeps the beam coherence intact. A third undulator with a magnetic period of 35 mm (U35) covers the full energy range of the instrument, from 8 keV to 20 keV (Narayanan *et al.*, 2018 ▸).

**Figure 1:**
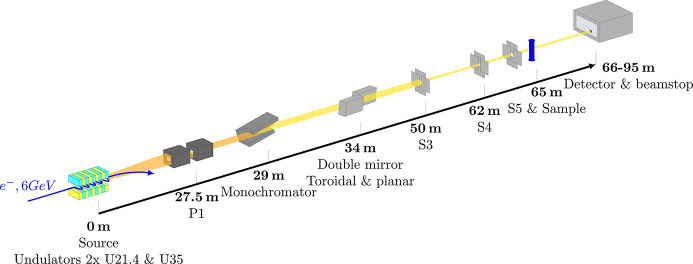
Beamline layout of the TRUSAXS instrument, with optical components used for XPCS measurements. From left to right: the insertion devices as X-ray source, P1 high-power slits, monochromator, double mirror, collimation slits S3 and S4, guard slits S5, sample, beamstop and detector. The orange line is the polychromatic beam whereas yellow represents the monochromatic beam.

Figure 1 ▸ shows the beamline layout with the main optical elements used for XPCS. The central cone of the undulator spectrum is selected by a pair of primary rectangular slits with an aperture of 150 µm × 150 µm (P1 and P2). The incident polychromatic beam is monochromated by a liquid-nitrogen-cooled channel-cut Si-111 crystal. The obtained energy dispersion is Δ*E*/*E* ≃ 1.5 × 10^−4^. After the monochromator, the beam is focused by a vertical toroidal mirror and steered parallel to the incident direction by a second planar mirror (Narayanan *et al.*, 2018 ▸). The horizontal focal point is about 33 m after the mirror (near the sample position), and the vertical focal point is usually 12 m inside the detector tube, depending on the angle of the mirror to the incident beam (Narayanan *et al.*, 2022 ▸). Further slit collimation is done using two pairs of slits 12 m apart from each other (S3 and S4). Since the beam is nearly coherent vertically and not on the transversal direction, these slits are opened to the beam size vertically, and to the size of a single coherence patch horizontally. The first slits of this pair diffract the beam, and the second select the central peak of the diffraction pattern. The aperture size for these slits is 15 µm horizontally and 40 µm vertically. Finally, just before the sample and 3 m after the second collimation slits, guard slits (S5) with aperture sizes of 160 µm × 140 µm (vertically × horizontally) clean the parasitic background. Beam images, shape and size near the sample and at the detector positions are given in the supporting information (SI). At the sample position, the coherent photon flux is of the order of 10^12^ photons s^−1^ at 12.230 keV. After the sample, the transmitted and scattered beams enter the 34 m-long evacuated flight tube toward the detector. The direct beam is stopped using a beamstop. To measure the sample transmission, p-i-n diodes (not shown in the layout) are used, one after S3 and the other just after the sample, on the entrance cone. Both of them use scattering from a window so that they do not block the direct beam.

### 2.2. Detectors

Two photon-counting pixel array detectors are available to perform XPCS measurements. The main one is an EIGER 500K consisting of eight sensitive areas of 256 × 256 pixels. It has been developed at the Paul Scherrer Institute, Switzerland, especially for high-throughput and high-frame-rate applications (maximum 23000 frames s^−1^ in 4-bit mode) (Dinapoli *et al.*, 2011 ▸). The performance of the EIGER 500K has been reported in an earlier publication (Zinn *et al.*, 2018 ▸). Most of the pixels are squares of 75 µm × 75 µm, except those at the edges of the sensitive areas, where the size is doubled vertically, horizontally or in both directions at the intersection of four sensitive areas. Those pixels can be masked by the acquisition software (*LIMA*).

The second detector used for XPCS is a commercial Dectris EIGER2 4M, composed of 2 × 4 modules, where each module is the same as the EIGER 500K detector (Donath *et al.*, 2023 ▸). The EIGER2 4M detector has a larger sensitive area (2068 × 2162 pixels) but is limited to 1000 frames s^−1^ in 8-bit mode. This is the detector used for (U)SAXS measurements (Narayanan *et al.*, 2022 ▸). Both EIGER detectors have a silicon sensor of thickness 450 µm, corresponding to an efficiency of about 80% at the standard operating energy of 12.230 keV.

These two detectors are mounted on a motorized table inside the wagon of the 34 m detector tube. This makes the detector change easy and allows a continuous variation of the sample-to-detector distance from 1.0 m to 31 m. A beamstop is inserted in front of the wagon and aligned with the incident beam to protect the detectors from direct exposure. Several beamstops are mounted on a frame and selected according to requirement (Narayanan *et al.*, 2018 ▸). By using the smallest circular beamstop (1 mm), at the standard energy, the *q*-range covered on the USAXS detector is from 1 × 10^−3^ nm^−1^ to 0.2 nm^−1^ at 31 m, where *q* is the modulus of the scattering vector, **q**, given by 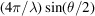 , and λ and θ are the X-ray wavelength and scattering angle, respectively. The speckle contrast increases with the sample-to-detector distance because of the better speckle resolution (see Section 4). Therefore, only distances from 15 m to 31 m are primarily used to perform XPCS measurements, limiting the upper *q*-range to about 0.7 nm^−1^.

### 2.3. Multispeckle XPCS

In an XPCS experiment, the quantity derived is the intensity–intensity autocorrelation function, *g*_2_(**q**, τ), which is related to the electric field–field autocorrelation function or intermediate scattering function, *g*_1_(**q**, τ), through the Siegert relation (Berne & Pecora, 2000 ▸), 

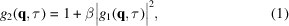

where β is the speckle contrast that depends on the coherence of the incoming X-ray beam, the counting accuracy of the detector and the angular resolution of the scattering setup. Here β is given by the square of the ratio of the variance and mean of the speckle intensity and therefore corresponds to the square of the usual definition of speckle contrast (Goodman, 1985 ▸). For the ideal case of a fully coherent incoming X-ray beam and speckle size larger than detector pixel size, β = 1. However, due to the limited coherence of the synchrotron source and detector resolution, this factor is usually smaller than 1 (Grübel *et al.*, 2008 ▸).

In a multispeckle XPCS experiment, a sequence of two-dimensional frames is recorded with exposure and lag times much shorter than the typical relaxation time probed in the sample. A key advantage of using a 2D detector to perform XPCS measurements is the ability to record multiple speckles for a given *q*, as well as orientation-dependent features (Fluerasu *et al.*, 2008 ▸; Möller & Narayanan, 2017 ▸; Pal *et al.*, 2018 ▸; Zinn *et al.*, 2023 ▸; Cheng *et al.*, 2021 ▸). These measurements allow calculating the ensemble-averaged *g*_2_(**q**, τ) with a single acquisition over a wide *q*-range (Lumma *et al.*, 2000 ▸). From the temporal fluctuations of the speckle pattern, the normalized ensemble-averaged two-time correlation function [TTCF; *g*_tt_(*q*, τ, *t*)] (Brown *et al.*, 1997 ▸) is derived, 

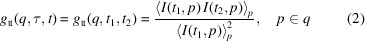

where *t*_1_ and *t*_2_ are the time of frames 1 and 2, respectively. τ = *t*_2_ − *t*_1_ is the lag time, *t* = (*t*_1_ + *t*_2_)/2 is the age, 〈…〉_*p*_ is the average over all pixels in the corresponding *q*-bin, and *I*(*t*, *p*) is the intensity of pixel *p* at time *t*. TTCF can be used to study out-of-equilibrium dynamics (Malik *et al.*, 1998 ▸). When the system is at (pseudo-)steady state, this TTCF can be time-averaged to obtain the standard *g*_2_(*q*, τ) (Brown *et al.*, 1997 ▸), 

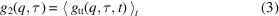

where 〈…〉_*t*_ is the average over all ages. *Dynamix* software also computes the standard deviation of *g*_2_(*q*, τ) and the normalized variance is directly related to the dynamical susceptibility, χ_4_(τ, *q*), that characterizes the steadiness of the system over the measurement (Duri & Cipelletti, 2006 ▸) (see also SI).

## 3. Data processing and visualization tools

### 3.1. Data processing

**Figure 2:**
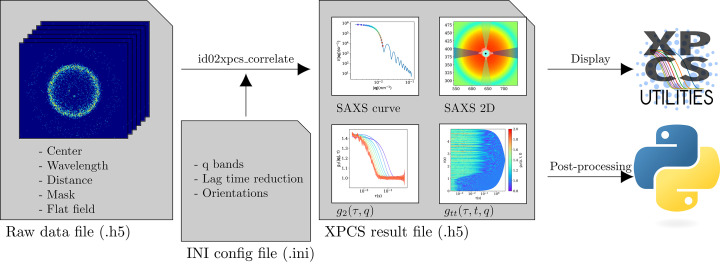
Schematic representation of the XPCS data processing workflow implemented. The processing starts from the raw data file, which contains the recorded patterns as well as metadata required for the calculation. The processing software *id02xpcs_correlate* performs the correlation according to the specifications in the INI file using the *Dynamix* package and writes the results in HDF5 format. The result file can be displayed using *XPCSUtilities* software or post-processed with custom scripts.

XPCS data processing is performed using an instrument-specific software in order to take advantage of the beamline metadata collection (Chèvremont, 2023*b* ▸), using the *Dynamix* library (Paleo *et al.*, 2021 ▸). Both are publicly available on their respective Git repositories. Figure 2 ▸ shows the data processing scheme. All the information regarding the experiment, such as the sample-to-detector distance, beam center, mask file, photon energy, *etc*., are written in the acquisition file as metadata. On the other hand, information needed for the processing, such as selection of *q*-bins and orientation to be analyzed, are specified in a single text configuration file in INI format. This allows the configuration file to be reused for many acquisition files, as soon as the data processing parameters are fixed. In addition, this configuration file can override parameters from the data file, to ease the reprocessing of the data with different parameters if needed (*e.g.* reprocessing with a modified data mask).

The processing software first computes the time-averaged pattern and its azimuthal average, corrected by flat-field, solid angle and sample transmission. Then, for each orientation to be analyzed, it computes first a *q*-mask which attributes a correlation index for each pixel. The intensity autocorrelation functions are calculated pixel by pixel and then averaged for all pixels in the same *q*-bin to obtain the ensemble averaged quantity (see Section 2.3). For each index, TTCF [*g*_tt_(*q*, τ, *t*)] is calculated, from which the *g*_2_(*q*, τ) function and its standard deviation are obtained by performing the time average. To ensure consistency of the results file, all results and computation parameters are saved into one HDF5 file, compliant with NeXus format (Könnecke *et al.*, 2015 ▸).

For online data processing, a lightweight server detects the new XPCS acquisition files and launches the data processing with a predefined list of INI configuration files. For most of the acquisitions, the correlation functions are available in less than one minute after measurement, which is a huge advantage compared with the previous XPCS data reduction procedure involving manual operations. Typical timing to perform the correlation on 35 narrow bins on a 10000 frames dataset in 8-bit mode is 13 s to read the data from the central storage, 1.2 s to perform SAXS azimuthal regrouping and other reduction steps, 3 s to initialize the correlator on the GPU, 16 s to compute all the TTCF and *g*_2_(*q*, *t*) functions with logarithmic distribution of lag time, and 5 s to save the results to disk. For the same dataset, if the logarithmic spacing of lag time is not performed, it take 84 s to save the results to disk.

### 3.2. Visualization tool

In addition to the processing software, a display tool, named *XPCSUtilities* (Chèvremont, 2023*a* ▸), has been developed to ease the visualization of the results immediately after the measurement. It is publicly available on the Git repository, and can be installed by any user on a Linux, Mac or Windows computer using the pip utility. The first function of this software is to visualize XPCS data on the fly. It has the ability to display the 2D SAXS pattern and the azimuthally regrouped curve, *g*_tt_(*q*, τ, *t*) and *g*_2_(*q*, τ), from the results file.

This software can also perform basic data processing, such as exporting *g*_2_(*q*, τ) as ASCII files. Some basic data analysis procedures are also implemented to evaluate the data and decide on the next measurements. There is a basic fitting tool, which fits the data with predefined functions. The last tool is a model-free relaxation time-Laplace analyzer, based on the multiscale CONTIN method (Liénard *et al.*, 2022 ▸), and adapted to work with multiple *q* values.

Additionally, this software can be used as a Python package to open and manipulate the XPCS results files, and used to script all of the tools described above. The online visualization and analysis tools hopefully aid non-expert users in more effective exploitation of the XPCS data.

### 3.3. Two-time correlation functions

The TTCF is often represented as a square matrix, where each element of the matrix (*TTCF*_*i*, *j*_) is the correlation amplitude of frame *i* at time *t*_1_ and frame *j* at time *t*_2_. This results in a huge matrix with *N* ^2^ elements, where *N* is the number of frames. By construction, this matrix is symmetric, the age axis is along the first diagonal and the lag time axis is along the second one (see Section 2.3 for definitions of age and lag time). On the other hand, it is usual for the *g*_2_(**q**, τ) function to display the lag time axis in logarithmic spacing, which allows visualizing all time scales at once, since the TTCF provides a very sensitive measure of the underlying dynamics and dynamic heterogeneities (Brown *et al.*, 1997 ▸; Lehmkühler *et al.*, 2021 ▸), and it is convenient to look at the dynamics in age/lag time coordinates (Perakis *et al.*, 2017 ▸). Therefore, the data are saved by default in these coordinates, with logarithmic spacing of the lag time. *XPCSUtilities* allows the TTCF to be displayed in any of these coordinates.

**Figure 3:**
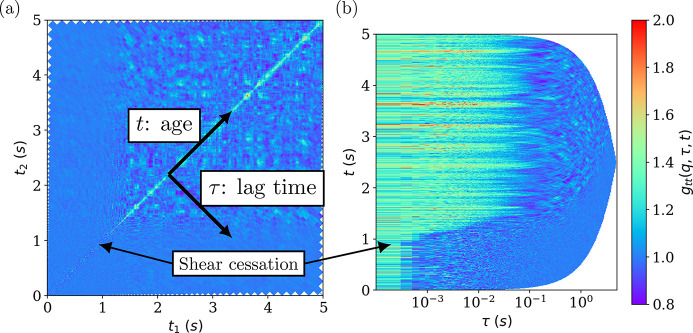
An example of the TTCF for a colloidal suspension subjected to uniform shear upon the cessation of flow in (*a*) (*t*_1_, *t*_2_) coordinates and (*b*) lag time/age coordinates, at *q* = 6 × 10^−3^ nm^−1^.

Figure 3 ▸ shows the TTCF captured during the shear cessation of a colloidal suspension consisting of silica particles with mean diameter 600 nm, (*a*) in *t*_1_, *t*_2_ coordinates and (*b*) in lag time-age coordinates. After approximately 1 s the rotor is stopped, leading to a clear change in the TTCF. In the first representation (*a*), most of the space is occupied by fluctuations around 1, and all the changes occur close to the diagonal, which is not very convenient to closely examine the transient features. In the second representation (*b*), the lag time has been represented in logarithmic scaling, so that all the time scales become visible. This representation can be directly seen as a stack of *g*_2_(**q**, τ) functions, evolving with the acquisition time (age axis) as traditionally represented in dynamic light scattering. As a result, the transition from advective to diffusive dynamics and subsequent evolution is clearly visible.

Additional advantages of the logarithmic lag time/age representation are the drastic reduction of the saving time as well as disk and memory consumption. This also allows the TTCFs to be saved more easily at each *q*, while maintaining a reasonable file size. A further benefit of this saving method is the ease to reslice and average in time the TTCF to obtain the evolution of the *g*_2_(**q**, τ) function. A case where this method is not recommended is when *g*_2_(**q**, τ) exhibits periodic features, for example as in XPCS-echo, where the sample is in an oscillatory motion (Leheny *et al.*, 2015 ▸).

## 4. Performance

To evaluate the improved performance of XPCS on the TRUSAXS instrument after the EBS upgrade, XPCS acquisitions under different conditions have been carried out. Unless specified otherwise, the standard energy of the beamline, 12.230 keV, has been used and the setup is as described in Section 2.1. For benchmarking XPCS, an aqueous suspension of nearly monodisperse (polydispersity below 2%) spherical silica particles with a diameter of 600 nm and volume fraction ϕ ≃ 0.01 was used. These silica particles were first resuspended in a quartz capillary of diameter 1 mm, and then kept at rest long enough to exhibit only Brownian behavior (Möller & Narayanan, 2017 ▸).

**Figure 4:**
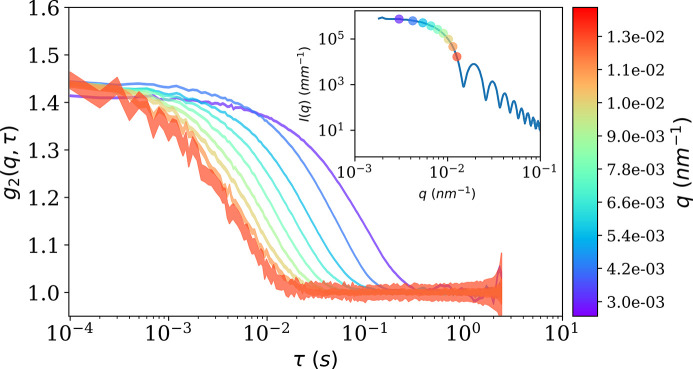
Measured *g*_2_(*q*, τ) from a suspension of silica particles with mean diameter of 600 nm and a volume fraction ϕ ≃ 0.01 in water at *T* = 298 K. The spread of a line is the calculated standard deviation. The inset shows the SAXS profile obtained by time-averaging the frames and then azimuthally regrouping. The points on the SAXS curve indicate where the *g*_2_(*q*, τ) functions have been computed, according to color.

Figure 4 ▸ shows the measured *g*_2_(*q*, τ) of the silica particle suspension displaying Brownian behavior. The inset shows points on the SAXS curve where the *g*_2_(*q*, τ) functions have been computed. The line spread is the standard deviation of the measurement. The SAXS profile displays the Guinier region and the oscillations of the spherical scattering form factor. Due to the high scattering power, the incident intensity was attenuated by a factor of about 30 to avoid detector saturation. In this figure, *g*_2_(*q*, τ) fully decays within the sampled time scale, for all accessible *q*. When the scattering intensity decreases, the detector receives fewer and fewer photons, and at higher *q* the standard deviation starts to be non-negligible. In practice, as the decay occurs in shorter lag time and *g*_2_(*q*, τ) becomes too noisy, it is difficult to make useful measurements at higher *q* with an attenuated beam.

### 4.1. Speckle contrast

The speckle contrast (β) is a sample-independent quantity (Abernathy *et al.*, 1998 ▸) defined by the experimental parameters such as the coherence of the incident X-ray beam and the resolution of speckles on the detector. Following the ideas of Sandy *et al.* (1999 ▸), Lumma *et al.* (2000 ▸) and Möller *et al.* (2019*a* ▸), at zero azimuth and when the sample is immediately adjacent to collimating slits (near field, λ*l*/*d* < *d*, *d* being the slit aperture and *l* the slits-to-sample distance), the contributions to β can be factorized into a two-dimensional integral [β_*z*_(*V*_*z*_, ζ)] and a four-dimensional integral [β_*xy*_(*q*, *V*_*x*_, *V*_*y*_, ξ, λ, Δ*E*/*E*)]. The first factor is related to the illuminated area on the *z*-axis, compared with the coherence on this axis. The second one is related to the illuminated area on the *xy*-plane, compared with the coherence on this plane. [β_*q*_(*q*, *S*_*y*_, *V*_*x*_, λ, Δ*E*/*E*)] expresses the contrast of the speckle tilted by the scattering angle (Hruszkewycz *et al.*, 2012 ▸). The two additional factors are due to the limited angular resolution of the detector, along *x* and *z* [β_res,*i*_(*V*_*i*_, *L*, λ, *P*), *i* = {*x*, *z*}], 

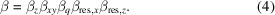

Here the axes coordinates are defined as follows: *y* – axis of the beam; *x* – horizontal axis perpendicular to the beam; *z* – vertical axis. The full expressions are described in the SI. The beamline-specific parameters used to compute the theoretical speckle contrast are provided in Table 1 ▸. All the values in Table 1 ▸ are determined by the storage ring and the beamline parameters.

**Figure 5:**
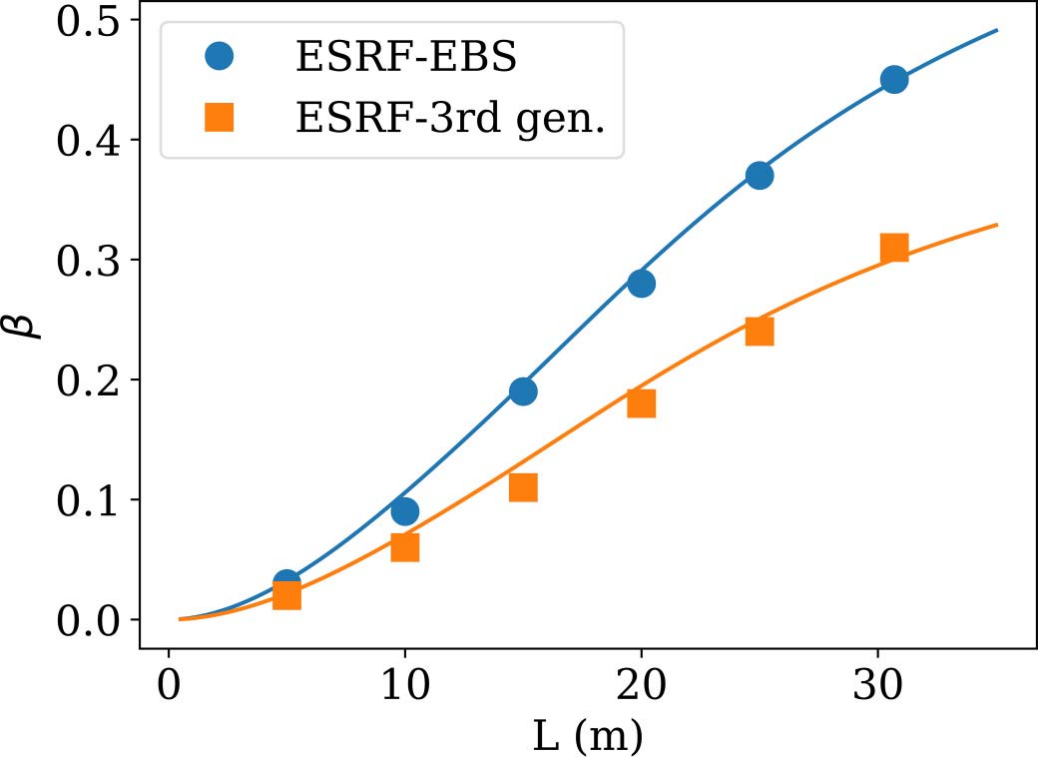
Experimentally obtained speckle contrast as a function of sample-to-detector distance measured using silica colloidal particles (600 nm in size) in water (*q* = 1.02 × 10^−2^ nm^−1^ at *E* = 12.230 keV), after the EBS upgrade. For a comparison the values before the upgrade are also shown. The lines are the theoretical values according to equation (4).

Figure 5 ▸ shows the measured speckle contrasts before and after the EBS upgrade, for the range of sample-to-detector distances feasible for XPCS. The points are the measured β values using a dilute colloidal suspension of silica particles (mean size 600 nm), whereas the curves are the prediction using equation (4). For both cases, before and after the EBS upgrade, the theoretical β value has been recovered on the sample tested. In addition, the EBS upgrade also improved the photon flux, sufficient to allow XPCS to be performed at higher energies.

**Figure 6:**
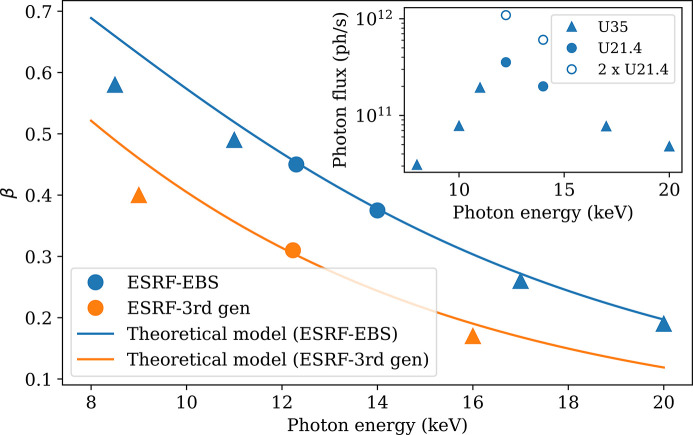
Variation of speckle contrast with photon energy (*q* = 1.02 × 10^−2^ nm^−1^ and *L* = 31 m). Points are measured values, continuous lines are theoretical prediction by equation (4) for the ESRF-EBS and former ESRF third-generation (3rd gen) storage ring. The inset shows the coherent photon flux available at the sample position with the EBS using different undulators.

Figure 6 ▸ shows the measured speckle contrast at different energies compared with the theoretical prediction. The measured values follow well the theoretical predictions by equation (4). The main reason why β is decreasing with photon energy is that the speckle size decreases with the photon energy, so that the speckles are less and less resolved by the detector pixels. The inset shows the photon flux at the sample position corresponding to different undulators of the beamline. The flux is the highest at 12.230 keV, since this is the fundamental energy of the U21.4 undulators at the minimum gap. For the other energies, the undulator harmonic has been chosen to maximize the photon flux and therefore the flux does not follow the expected λ^2^ dependence (Grübel *et al.*, 2008 ▸).

**Figure 7:**
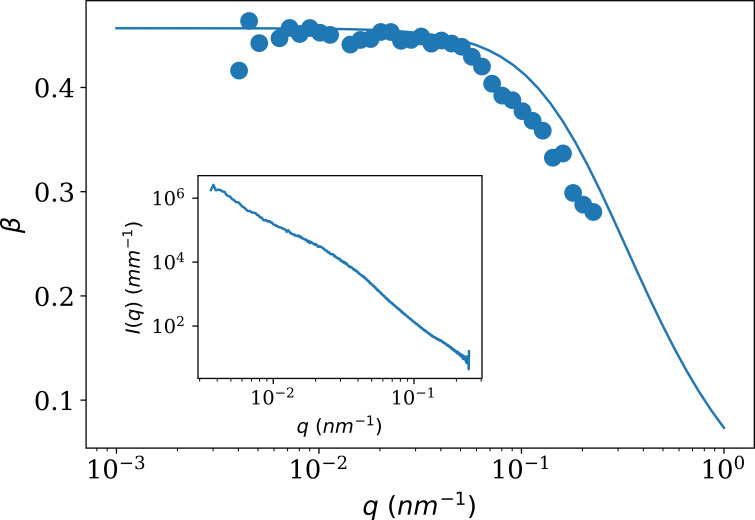
Variation of the speckle contrast with scattering vector. Points are measured speckle contrasts using Al_2_O_3_ powder. The continuous line is the theoretical curve according to equation (4). The inset shows the background-subtracted SAXS profile of the alumina powder.

Figure 7 ▸ shows the variation of speckle contrast with the scattering vector, *q* (*E* = 12.230 keV and *L* = 31 m). The points are measured using static speckles from Al_2_O_3_ powder, whose SAXS curve is shown in the inset. The continuous line is the theoretical curve. The decrease at high *q* originates mainly from β_*q*_, which takes into account the tilt of the speckles by the scattering angle (Hruszkewycz *et al.*, 2012 ▸).

### 4.2. Speckle contrast reduction at high count rates

This section shows the speckle contrast reduction related to various artifacts such as detector saturation, counter overflow and signal/background ratio.

**Figure 8:**
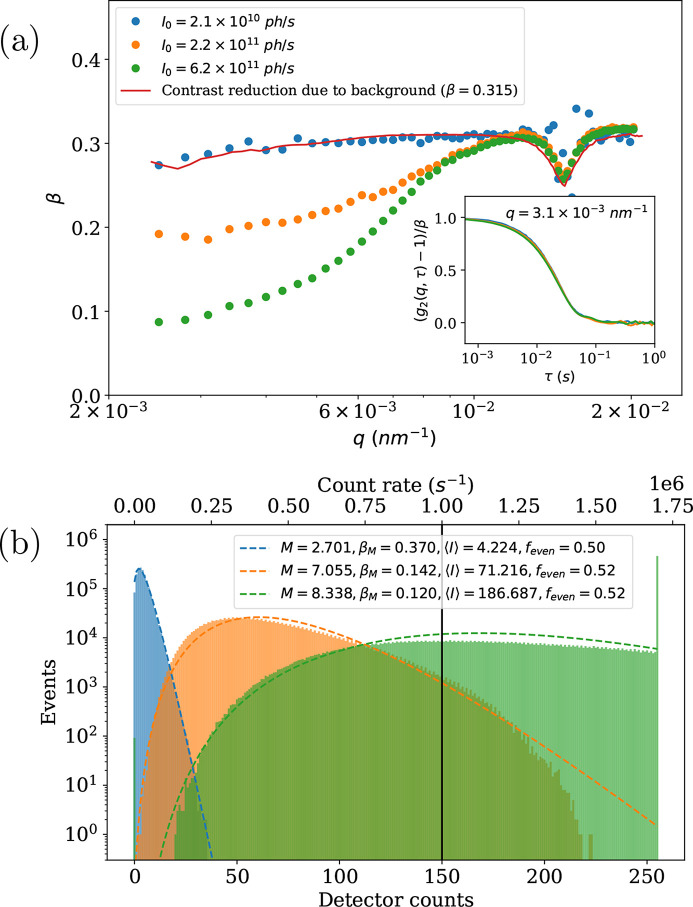
(*a*) Variation of speckle contrast with *q*. The insert shows the rescaled *g*_2_(*q*, τ) by removing 1 and dividing by the contrast at *q* = 3.1 × 10^−3^ nm^−1^. (*b*) Histograms of pixel intensities (counts) at *q* = 3.1 × 10^−3^ nm^−1^. Dashed lines are the fits to the Poisson–gamma distribution function [equation (5)]. The sample is a dilute colloidal suspension of silica particles (600 nm in diameter) at rest, measured with different incident beam intensities. The measurements were performed using a larger beam that led to a maximum contrast of 0.315.

Figure 8 ▸(*a*) shows the evolution of speckle contrast as a function of *q*. The sample is a similar silica colloidal suspension shown in the inset of Fig. 4 ▸. The unsubtracted SAXS profile of the sample and background are shown in Fig. 3 of SI. The incident flux increased from blue to green by removing an attenuator and inserting a second undulator. For the curves measured with larger incident flux, β is clearly reduced in the Guinier region, where the scattering intensity is the highest. β then increased as the count rate on the detector decreased at larger *q* range. Note that this is not a beam-induced effect as the morphology of the particles is extremely stable, the structure factor of the interactions is negligible and beam heating is insignificant. Moreover, this problem does not arise if the sample is diluted 30 times with correspondingly lower scattering power (Zinn *et al.*, 2022 ▸).

A likely source of this effect is the artifacts in photon-counting statistics introduced by the detector hardware or the specific dead-time correction procedure implemented (Zambon, 2021 ▸). At *q* = 1.47 × 10^−2^ nm^−1^ the speckle contrast exhibits the same minimum for all three incident intensities. This minimum corresponds to the first minimum in the scattering form factor (Fig. 4 ▸). In this region, the background scattering and sample scattering become closer, as shown by the SAXS profile in Fig. 3 of SI. This reduces β by a factor of (1 + *X*)^2^, where *X* is the ratio of background to sample scattering intensity (Lhermitte *et al.*, 2017 ▸; Chushkin, 2023 ▸). This factor can be obtained directly by considering a constant background intensity (*I*_B_) and computing the correlation function on 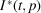 = *I*(*t*, *p*) + *I*_B_(*p*) instead of *I*(*t*, *p*). The red curve in Fig. 8 ▸(*a*) is this factor obtained from the SAXS curve of the sample and background, multiplied by a constant β value. The inset of Fig. 8 ▸(*a*) shows the rescaled *g*_2_(*q*, τ) at *q* = 3.1 × 10^−3^ nm^−1^. Despite the variation of β values among different measurements, nearly the same relaxation rate is preserved.

Figure 8 ▸(*b*) shows histograms of the pixel counts (intensities) used to compute the multispeckle *g*_2_(*q*, τ) functions at *q* = 3.1 × 10^−3^ nm^−1^. The dashed lines are the fits by a negative binomial or Poisson–Gamma distribution function [*P*_*M*_(*I*)] (Goodman, 1985 ▸), 

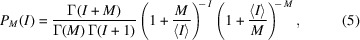

where *I* is the detector counts and *M* is the number of modes. β_*M*_ = 1/*M* is the contrast computed from the number of modes. Additional histograms for *q* = 1 × 10^−2^ nm^−1^ are shown in Fig. 2 of SI.

From these histograms it is apparent that, in the curve with highest flux (green), many pixels are saturated at 255, the maximum value in 8-bit mode. Additionally, the count rate on these pixels exceeds 10^6^ s^−1^, where the non-linearity tends to appear when measuring SAXS patterns (pileup effect) (Donath *et al.*, 2023 ▸). For the orange symbols, there is no counter overflow (<255), and most of the pixel counts are below the limit of 10^6^ s^−1^. However, the speckle contrast is still decreased. On the histogram, a crenellation pattern is visible, which indicates that there are more even values than odd values. There is no reason why this should happen with this large number of events and such a wide distribution. A similar effect has already been seen with the EIGER detector at low counts (Möller *et al.*, 2019*b* ▸), where the even and odd bytes were swapped.

Additionally, the distribution of detector counts deviates from the Poisson–Gamma distribution for the orange and green curves. So, this effect, together with the close-to-limit count rate, reduces the β values of these curves. Finally, the blue curves show only a weak reduction of β at low *q*, that can be explained by the background/signal ratio. This has been recorded with a maximum count rate of 2.5 × 10^5^ s^−1^, far away from the counter overflow and count rate saturation (pileup). The histogram shows a Poisson–Gamma distribution and does not display a crenellation pattern, which is the ideal case.

### 4.3. Measurement of stability

The stability of the TRUSAXS beamline, and thus its ability to perform long-time XPCS measurements, has been characterized by recording static speckles generated by a thin layer of alumina powder exposed to the coherent beam. The speckle pattern has been recorded for 8 h and 12 h every 15 s and 30 s, respectively.

**Figure 9:**
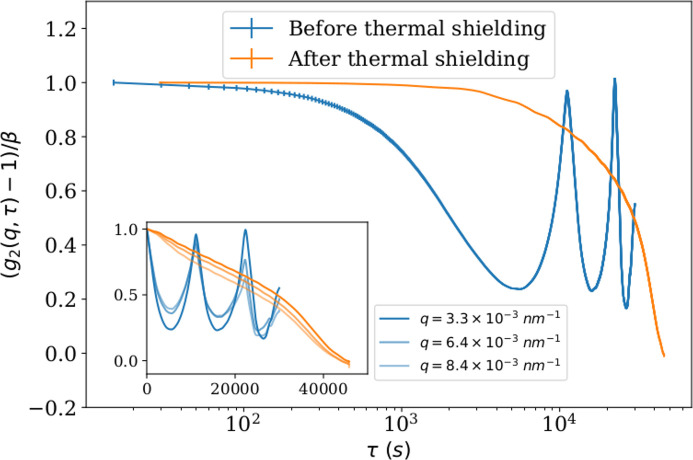
Normalized *g*_2_(*q*, τ) of an alumina powder sample, at *q* = 3.3 × 10^−3^ nm^−1^, before and after thermal shielding of the support of the S4 slits. The inset shows the same curves at different *q*, with linear scaling of the *x*-axis.

Figure 9 ▸ shows the normalized *g*_2_(*q*, τ) recorded with an alumina powder sample. The alumina powder has been immobilized on a tape and fixed firmly on the sample holder, so that the sample itself is perfectly static, and thus cannot induce any decorrelation of *g*_2_(*q*, τ). The inset shows the same curves at different *q*, with linear scaling of the lag time axis. It shows that there is no *q*-dependence, and therefore the decorrelation is more likely to arise from a bulk sample movement. The blue curve shows oscillations, with a period of 11000 s (about 3 h). With this setup, only measurements below 100 s were feasible without being affected by this instability. After investigations, it has been found that this oscillatory motion (echos) came from the last collimation slits (S4). These slits are mounted on a cast iron pillar, which is then sensitive to thermal fluctuations inside the experimental hutch. This pillar has now been shielded with a wooden box in order to reduce the temperature variations around it. The orange curve was measured after the pillar shielding. It exhibits now a plateau up to 3000 s (50 min), which extends the accessible measurement time to this value, until a decorrelation from the instrument is affecting the measurements. The remaining decay is likely related to thermal expansion of the sample table itself, which induces a relative motion of the sample on a time scale of hours. An X-ray beam-induced grain motion (Shinohara *et al.*, 2015 ▸) is excluded in this case, since the sample is not continuously exposed to the beam.

## 5. Scientific applications

This section presents two representative applications of XPCS using the TRUSAXS instrument. Several recent examples are presented elsewhere (Zinn *et al.*, 2022 ▸, 2023 ▸; Pal *et al.*, 2022 ▸; Narayanan *et al.*, 2023 ▸).

### 5.1. Rheo-XPCS: transient dynamics following the cessation of shear

This experiment was performed using a Haake RS6000 (ThermoFischer Scientific) rheometer. The rheological cell consists of in-house-developed Searle-type coaxial capillaries (Narayanan *et al.*, 2020 ▸). The stator is a 2 mm inner-diameter quartz capillary and the rotor is a 1 mm outer-diameter quartz capillary. A scheme of the capillary shear cell used is shown in Fig. 4 of SI. The fluid under study was a dilute suspension of silica particles, 600 nm in diameter, in water. In this experiment the suspension is stirred using the rheometer, and the X-ray beam passed just below the rotor, 0.5 mm from the tip. This configuration allows the suspension to be stirred by the rotor, but does not generate a uniform shear flow, which would tend to homogenize the suspension.

The sample was stirred at a constant shear rate (200 s^−1^) and then the rotor was stopped suddenly. XPCS acquisitions were started before the cessation of flow, a long one while the rotor was stopped and then every 5 minutes until the suspension recovered the Brownian behavior. In this experimental configuration it is clear that the dynamics in the vertical and horizontal directions are different. The vertical direction has contributions from both Brownian motion and sedimentation. In the horizontal direction both Brownian motion and inertia from the shear flow contribute to the decay of *g*_2_(**q**, τ). Therefore, *g*_2_(**q**, τ) functions need to be computed separately in the vertical and horizontal directions.

The functional form of *g*_2_(**q**, τ) has to take into account the two contributions identified earlier: the Brownian diffusion and advection due to the flow and sedimentation. The term for Brownian diffusion is well established (Berne & Pecora, 2000 ▸). On the other hand, the function form due to fluid motion strongly depends on the exact shape of the velocity distribution (Tong *et al.*, 1988 ▸). Because of the homodyne detection scheme, the measured *g*_2_(**q**, τ) functions are only sensitive to velocity fluctuations and the mean velocity enters as a *q*-independent transit term (Leheny *et al.*, 2015 ▸).

**Figure 10:**
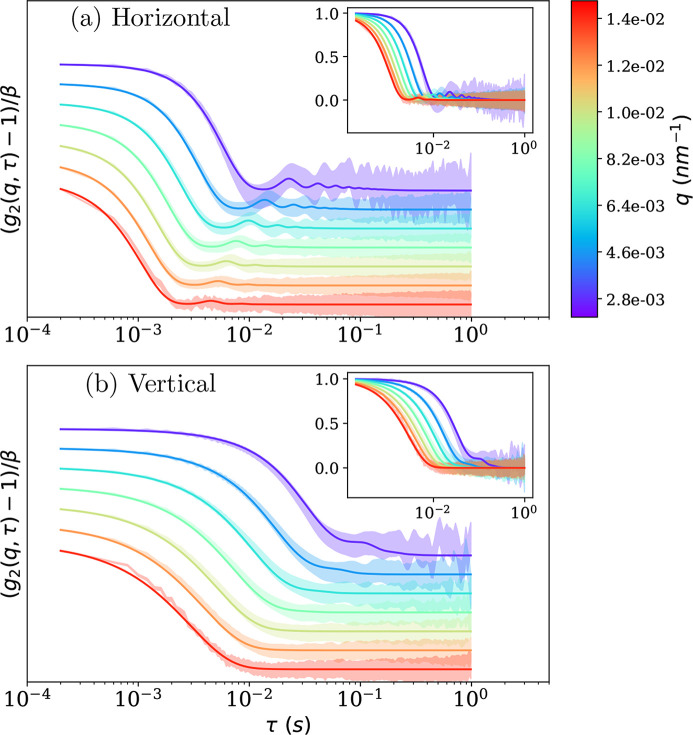
Measured *g*_2_(**q**, τ) of a dilute silica colloidal suspension (mean diameter ∼600 nm) following the cessation of shear. The shaded area represents experimental *g*_2_(**q**, τ) whose height is the standard deviation and lines are the fit to equation (6). For clarity, the curves have been vertically shifted. The inset shows curves without this shift.

Examination of the data in Fig. 10 ▸ clearly shows oscillations in *g*_2_(**q**, τ). This is a signature of constant velocity differences, which leads to a sinc function (Möller & Narayanan, 2017 ▸). The equation that combines Brownian diffusion, advection and transit terms can be written as (Leheny *et al.*, 2015 ▸; Zinn *et al.*, 2020 ▸)

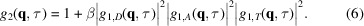

The diffusive term, *g*_1,*D*_, can be written as (Berne & Pecora, 2000 ▸) 

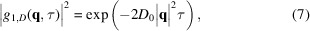




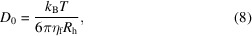

where *D*_0_ is the Stokes–Einstein diffusion coefficient, *R*_h_ is the hydrodynamic radius, *k*_B_ is the Boltzmann constant, η_f_ is the fluid viscosity and *T* is the absolute temperature. The advective term is given by (Möller & Narayanan, 2017 ▸) 

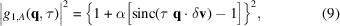

where δ*v* is the mean velocity fluctuation and α represents the fraction of particles deviating from the mean velocity (*v*). Finally, the transit term is given by (Weber & Schweiger, 1998 ▸) 

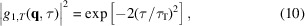

where τ_T_ is the transit time, τ_T_ = σ_B_/*v*, with σ_B_ the Gaussian width of the beam.

Equation (6) has four free parameters to be fitted: β, *D*_0_, α and δ*v*. β needs to be adjusted to each single curve, whereas the three other parameters are *q*-independent. Since the shape of *g*_2_(**q**, τ) is only weakly affected by the parameters compared with β, direct fitting of all curves with these parameters did not give a satisfactory result. Most of the changes occur in the slope of the decay, which was never well described by the direct fitting procedure, even when weighted by the first derivative (most of the points before and after the decay are constant and equal to β or 0). To circumvent this problem, an iterative fitting procedure has been adopted.

β was first rescaled by fitting the initial decay with an exponential function, in order to obtain an initial guess. The rescaled curves were fitted with the *q*-dependent equation. β of each curve was then adjusted to better match the fitted function. The second and third steps were repeated until the procedure converged. This iterative procedure gave more satisfactory results and has been found to be stable for a wide range of initial parameters. Figure 10 ▸ compares the results of this fitting procedure with the experimental data. By this iterative procedure, the data (shaded area) are well described by the fit function, especially the decay and the first oscillation when visible. Even in the vertical direction [Fig. 10 ▸(*b*)], where the oscillations are very weak, the decay is still well described.

The above procedure has been first applied to the whole data set, from the shear cessation instant to the final Brownian state, with three free parameters. At this stage, parameters *D*_0_ and α did not show any systematic changes with time, and remained constant within the uncertainties. The average value of *D*_0_ was very close to the theoretical one (0.85 µm^2^ s^−1^). Similarly, the average value of α was very close to 0.5. To improve the fits, these values have been fixed, leaving only β and δ*v* as adjustable parameters. With the reduced number of parameters, the fits were still very good at describing the data over the whole *q*-range, but this significantly improved the temporal decay of δ*v*.

**Figure 11:**
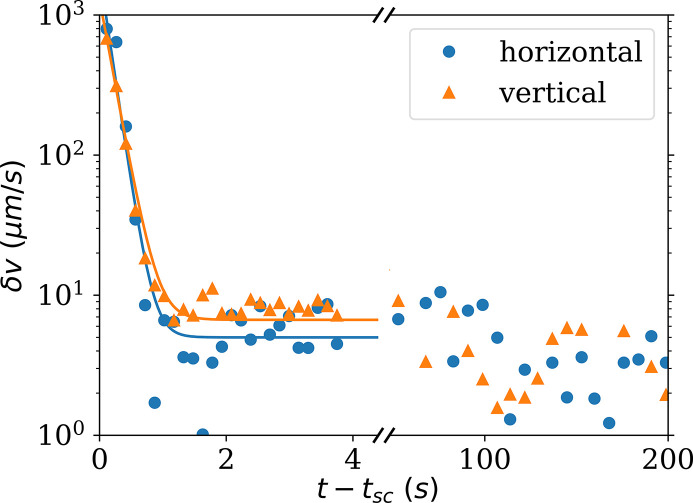
Decay of mean velocity fluctuations in the vertical and horizontal directions, after stopping the rotor (*t*_sc_). The initial shear rate was 200 s^−1^ and the beam traversed 0.5 mm below the rotor. Time is taken relative to the shear cessation.

To obtain the evolution of *g*_2_(**q**, τ) just after the cessation of shear, the TTCF functions taken while the rotor stopped were resliced and averaged (see Section 3.3). Figure 11 ▸ shows the decay of δ*v* below the rotor, just after the cessation of shear. A large number of points have been acquired just after the cessation of shear, thanks to the two-time correlation function. Just after the cessation of shear, δ*v* decays exponentially within a second and then decreases slowly at a different rate. Figure 3 ▸ depicted the TTCF during the cessation of shear. At the beginning, the shear rate is too rapid to be captured by the maximum frame rate of the detector (23 kHz), then there is a rapid increase of the relaxation time (which corresponds to a rapid decrease of δ*v*), then a slower decay. The Brownian limit is reached after several minutes. This behavior is systematic but depends on the initial shear rate. This example illustrates the ability to probe relatively fast out-of-equilibrium dynamics by XPCS.

### 5.2. High-pressure XPCS

To illustrate the feasibility of performing XPCS at higher energy, where the speckle contrast and photon flux are lower, the diffusion coefficient of sterically stabilized PMMA colloids of mean radius *R*_S_ = 380 nm in *cis*-decalin was measured using a high-pressure cell at an X-ray energy of 17 keV. This high-pressure cell (Möller *et al.*, 2016 ▸) has two diamond windows, each 1 mm thick, which guides the choice of higher energy to allow sufficient beam transmission through the cell. The pressure was varied from 0.1 MPa to 140 MPa, which increased the viscosity of *cis*-decalin by a factor of 4.2 (Zéberg-Mikkelsen *et al.*, 2003 ▸).

**Figure 12:**
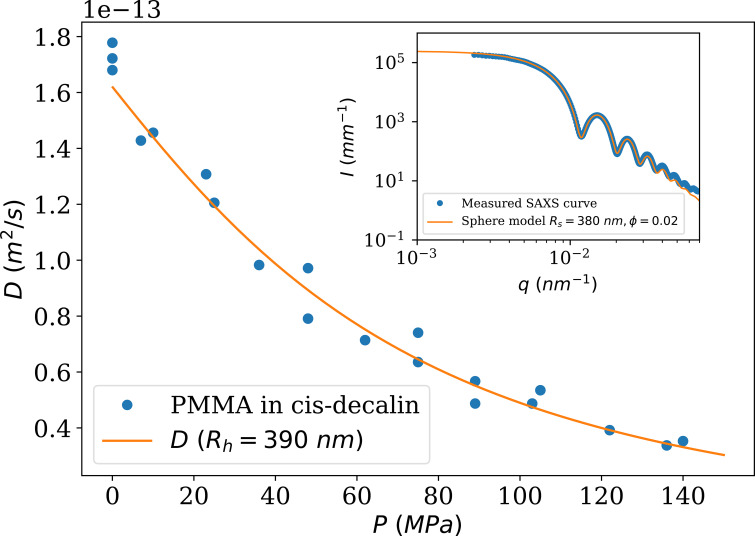
Evolution of the diffusion coefficient of colloidal particles of PMMA with mean core radius *R*_S_ = 380 nm in *cis*-decalin (ϕ ≃ 0.1). The points have been measured using XPCS and the line is the theoretical estimate. Temperature was set to 293 K. The inset shows the SAXS profile of a dilute sample (ϕ ≃ 0.02) together with a polydisperse sphere model.

Figure 12 ▸ shows the variation of the diffusion coefficient, *D*, of PMMA particles in *cis*-decalin. The points have been deduced from XPCS measurements, by fitting *g*_2_(τ, *q*) to an exponential function [see equations (7) and (8)]. The fitting procedure is the same as that described in Section 5.1. The continuous line was calculated using the Stokes–Einstein relation [equation (8)], considering the pressure dependence of viscosity taken from the literature (Zéberg-Mikkelsen *et al.*, 2003 ▸) and the evolution of the suspension viscosity with ϕ (Segrè *et al.*, 1995 ▸). The data obtained are in good agreement with the theoretical estimates for ϕ ≃ 0.12. This demonstrates the feasibility of such measurements, which were challenging to perform earlier because of the lack of flux and small speckle contrast at this higher energy (Moron *et al.*, 2022 ▸; Zhang *et al.*, 2023 ▸). This opens new opportunities for the XPCS technique with this kind of sample environments and samples which require use of higher photon energies.

## 6. Conclusion

The higher degree of coherence of the EBS together with fast pixel detectors have significantly improved the technical performance of the XPCS method (Chushkin *et al.*, 2022 ▸; Narayanan *et al.*, 2023 ▸). The UA-XPCS technique on the TRUSAXS instrument shows these benefits in terms of an enhanced speckle contrast and an order of magnitude higher coherent flux. The theoretical predictions of speckle contrast both with the old storage ring and EBS parameters are in good agreement with the measured values. With the next generation of pixel detectors (Nakaye *et al.*, 2021 ▸; Williams *et al.*, 2022 ▸), even faster time scales will be accessible by UA-XPCS.

The available flux at 12.230 keV enables performing XPCS measurements on dilute samples with reasonably good scattering contrast and probe relatively fast dynamics. The use of higher X-ray energy helps to reduce radiation damage with biological samples and soft matter (Möller *et al.*, 2019*a* ▸). If needed, XPCS measurements can be performed even at higher energies, ∼20 keV, with a speckle contrast of 20%. This opens new perspectives for XPCS studies of highly absorbing samples and sample environments, such as a high-pressure cell that has thick diamond windows (Moron *et al.*, 2022 ▸; Zhang *et al.*, 2023 ▸).

To better exploit the instrumental developments, a new software suite that enables online processing of XPCS data acquisition as well as the visualization of processed data has been developed. This significantly improves the user experience when performing XPCS using the TRUSAXS instrument, and hopefully helps in promoting the application of XPCS to new scientific cases. The detector is still a limitation when operating at both higher and lower count rates due to possible distortion of the counting statistics (Möller *et al.*, 2019*b* ▸).

Several examples of out-of-equilibrium dynamics probed by the XPCS technique at the TRUSAXS instrument have been reported (Zinn *et al.*, 2022 ▸; Narayanan *et al.*, 2023 ▸). The previous section presented additional cases demonstrating the enhanced performance. The shear cessation experiment illustrates fast multispeckle XPCS involving direction-dependent analysis. The second example shows the feasibility of XPCS in combination with a high-pressure cell, which has been limited before because of the lower flux and speckle contrast at higher energies. A broad scattering vector range and combination with a variety of sample environments broaden the scope of the XPCS for probing the dynamics in complex fluids.

## 7. Related literature

The following references, not cited in the main body of the paper, have been cited in the supporting information: Conrad *et al.* (2015 ▸); Dasgupta *et al.* (1991 ▸); Glotzer *et al.* (2000 ▸).

## Supplementary Material

Supporting Information (SI) providing the beam profile, speckle contrast calculation, additional intensity statistics, SAXS profile showing the background effect and the capillary shear cell setup. DOI: 10.1107/S1600577523008627/ju5056sup1.pdf

## Figures and Tables

**Table 1:** Main beamline parameters in coherent scattering mode used for calculation of the theoretical speckle contrast values

		ESRF	
Symbol	Description	3rd gen	EBS
*P*	Pixel size	0.075 mm	
*R*	Source-to-sample distance	65 m	
*S*_*i*_ = λ*L*/*V*_*i*_	Speckle size		
*L*	Sample-to-detector distance	1.5–30.7 m	
*V*	Illuminated volume along *x*, *y*, *z*	25 × 1000 × 30 µm	
σ_*x*_	Effective source size along *x*	128 µm	70 µm
σ_*z*_	Effective source size along *z*	11 µm	9 µm
ξ = λ*R*/(2πσ_*x*_)	Coherence length along *x*	8.2 µm	14.9 µm
ζ = λ*R*/(2πσ_*z*_)	Coherence length along *z*	95 µm	116 µm
λ	Photon wavelength	0.1 nm	
Δ*E*/*E*	Energy dispersion	1.5 × 10^−4^	

## References

[bb1] Abernathy, D. L., Grübel, G., Brauer, S., McNulty, I., Stephenson, G. B., Mochrie, S. G. J., Sandy, A. R., Mulders, N. & Sutton, M. (1998). *J. Synchrotron Rad.* **5**, 37–47. 10.1107/S0909049597015835 16687799

[bb2] Berne, B. J. & Pecora, R. (2000). *Dynamic Light Scattering: With Applications to Chemistry, Biology, and Physics.* Courier Corporation.

[bb3] Brown, G., Rikvold, P. A., Sutton, M. & Grant, M. (1997). *Phys. Rev. E*, **56**, 6601–6612.

[bb4] Cheng, C.-H., Kamitani, K., Masuda, S., Uno, K., Dechnarong, N., Hoshino, T., Kojio, K. & Takahara, A. (2021). *Polymer*, **229**, 124003.

[bb6] Chèvremont, W. (2023*a*). *XPCSUtilities*, https://gitlab.esrf.fr/id02/xpcsutilities.

[bb5] Chèvremont, W. (2023*b*). *id02xpcs*, https://gitlab.esrf.fr/id02/id02xpcs.

[bb7] Chushkin, Y. (2023). Private communication.

[bb8] Chushkin, Y., Gulotta, A., Roosen-Runge, F., Pal, A., Stradner, A. & Schurtenberger, P. (2022). *Phys. Rev. Lett.* **129**, 238001. 10.1103/PhysRevLett.129.238001 36563210

[bb842] Conrad, H., Lehmkühler, F., Fischer, B., Westermeier, F., Schroer, M. A., Chushkin, Y., Gutt, C., Sprung, M. & Grübel, G. (2015). *Phys. Rev. E*, **91**, 042309. 10.1103/PhysRevE.91.042309 25974493

[bb843] Dasgupta, C., Indrani, A. V., Ramaswamy, S. & Phani, M. K. (1991). *Europhys. Lett.* **15**, 307–312.

[bb9] Dinapoli, R., Bergamaschi, A., Henrich, B., Horisberger, R., Johnson, I., Mozzanica, A., Schmid, E., Schmitt, B., Schreiber, A., Shi, X. & Theidel, G. (2011). *Nucl. Instrum. Methods Phys. Res. A*, **650**, 79–83.

[bb10] Donath, T., Šišak Jung, D., Burian, M., Radicci, V., Zambon, P., Fitch, A. N., Dejoie, C., Zhang, B., Ruat, M., Hanfland, M., Kewish, C. M., van Riessen, G. A., Naumenko, D., Amenitsch, H., Bourenkov, G., Bricogne, G., Chari, A. & Schulze-Briese, C. (2023). *J. Synchrotron Rad.* **30**, 723–738. 10.1107/S160057752300454X PMC10325006 37343017

[bb11] Duri, A. & Cipelletti, L. (2006). *Europhys. Lett.* **76**, 972–978.

[bb12] Eriksson, M., van der Veen, J. F. & Quitmann, C. (2014). *J. Synchrotron Rad.* **21**, 837–842. 10.1107/S1600577514019286 25177975

[bb13] Fluerasu, A., Moussaïd, A., Falus, P., Gleyzolle, H. & Madsen, A. (2008). *J. Synchrotron Rad.* **15**, 378–384. 10.1107/S0909049508006420 18552431

[bb14] Goodman, J. W. (1985). *J. Opt. Soc. Am. A*, **2**, 1448–1454.

[bb844] Glotzer, S. C., Novikov, V. N. & Schrøder, T. B. (2000). *J. Chem. Phys.* **112**, 509–512.

[bb15] Grübel, G., Madsen, A. & Robert, A. (2008). *Soft Matter Characterization*, ch. 18. Berlin: Springer.

[bb16] Hruszkewycz, S. O., Sutton, M., Fuoss, P. H., Adams, B., Rosenkranz, S., Ludwig, K. F., Roseker, W., Fritz, D., Cammarata, M., Zhu, D., Lee, S., Lemke, H., Gutt, C., Robert, A., Grübel, G. & Stephenson, G. B. (2012). *Phys. Rev. Lett.* **109**, 185502. 10.1103/PhysRevLett.109.185502 23215295

[bb17] Könnecke, M., Akeroyd, F. A., Bernstein, H. J., Brewster, A. S., Campbell, S. I., Clausen, B., Cottrell, S., Hoffmann, J. U., Jemian, P. R., Männicke, D., Osborn, R., Peterson, P. F., Richter, T., Suzuki, J., Watts, B., Wintersberger, E. & Wuttke, J. (2015). *J. Appl. Cryst.* **48**, 301–305. 10.1107/S1600576714027575 PMC4453170 26089752

[bb18] Leheny, R. L., Rogers, M. C., Chen, K., Narayanan, S. & Harden, J. L. (2015). *Curr. Opin. Colloid Interface Sci.* **20**, 261–271.

[bb19] Lehmkühler, F., Roseker, W. & Grübel, G. (2021). *Appl. Sci.* **11**, 6179.

[bb553] Lhermitte, J. R. M., Rogers, M. C., Manet, S. & Sutton, M. (2017). *Rev. Sci. Instrum.* **88**, 015112. 10.1063/1.4974099 28147652

[bb20] Liénard, F., Freyssingeas, E. & Borgnat, P. (2022). *J. Chem. Phys.* **156**, 224901. 10.1063/5.0088005 35705415

[bb21] Liu, L., Alves, M., de Sá, F., Farias, R., Marques, S., Oliveira, A., Resende, X., Seraphim, R. & Westfahl, H. Jr (2022). *Proceedings of the 13th International Particle Accelerator Conference (IPAC2022)*, 12–17 June 2022, Bangkok, Thailand, pp. 1385–1388. TUPOMS002.

[bb22] Lumma, D., Lurio, L. B., Mochrie, S. G. J. & Sutton, M. (2000). *Rev. Sci. Instrum.* **71**, 3274–3289.

[bb23] Malik, A., Sandy, A., Lurio, L., Stephenson, G., Mochrie, S., McNulty, I. & Sutton, M. (1998). *Phys. Rev. Lett.* **81**, 5832–5835.

[bb24] Möller, J., Léonardon, J., Gorini, J., Dattani, R. & Narayanan, T. (2016). *Rev. Sci. Instrum.* **87**, 125116. 10.1063/1.4972296 28040915

[bb25] Möller, J. & Narayanan, T. (2017). *Phys. Rev. Lett.* **118**, 198001. 10.1103/PhysRevLett.118.198001 28548515

[bb26] Möller, J., Reiser, M., Hallmann, J., Boesenberg, U., Zozulya, A., Rahmann, H., Becker, A.-L., Westermeier, F., Zinn, T., Zontone, F., Gutt, C. & Madsen, A. (2019*b*). *J. Synchrotron Rad.* **26**, 1705–1715. 10.1107/S1600577519006349 31490162

[bb27] Möller, J., Sprung, M., Madsen, A. & Gutt, C. (2019*a*). *IUCrJ*, **6**, 794–803. 10.1107/S2052252519008273 PMC6760446 31576213

[bb28] Moron, M., Al-Masoodi, A., Lovato, C., Reiser, M., Randolph, L., Surmeier, G., Bolle, J., Westermeier, F., Sprung, M., Winter, R., Paulus, M. & Gutt, C. (2022). *J. Phys. Chem. B*, **126**, 4160–4167. 10.1021/acs.jpcb.2c01947 35594491

[bb29] Nakaye, Y., Sakumura, T., Sakuma, Y., Mikusu, S., Dawiec, A., Orsini, F., Grybos, P., Szczygiel, R., Maj, P., Ferrara, J. D. & Taguchi, T. (2021). *J. Synchrotron Rad.* **28**, 439–447. 10.1107/S1600577520016665 PMC7941290 33650555

[bb30] Narayanan, T., Chèvremont, W. & Zinn, T. (2023). *J. Appl. Cryst.* **56**, 939–946. 10.1107/S1600576723004971 PMC10405582 37555224

[bb31] Narayanan, T., Dattani, R., Möller, J. & Kwaśniewski, P. (2020). *Rev. Sci. Instrum.* **91**, 085102. 10.1063/5.0012905 32872916

[bb32] Narayanan, T. & Konovalov, O. (2020). *Materials*, **13**, 752. 10.3390/ma13030752 PMC7040635 32041363

[bb33] Narayanan, T., Sztucki, M., Van Vaerenbergh, P., Léonardon, J., Gorini, J., Claustre, L., Sever, F., Morse, J. & Boesecke, P. (2018). *J. Appl. Cryst.* **51**, 1511–1524. 10.1107/S1600576718012748 PMC6276275 30546286

[bb34] Narayanan, T., Sztucki, M., Zinn, T., Kieffer, J., Homs-Puron, A., Gorini, J., Van Vaerenbergh, P. & Boesecke, P. (2022). *J. Appl. Cryst.* **55**, 98–111. 10.1107/S1600576721012693 PMC8805168 35145357

[bb35] Pal, A., Kamal, M. A. & Schurtenberger, P. (2022). *J. Colloid Interface Sci.* **621**, 352–359. 10.1016/j.jcis.2022.04.063 35468558

[bb36] Pal, A., Zinn, T., Kamal, M. A., Narayanan, T. & Schurtenberger, P. (2018). *Small*, **14**, 1802233. 10.1002/smll.201802233 30102453

[bb37] Paleo, P., Kieffer, J. & Chushkin, Y. (2021). *silx-kit/dynamix: Dynamix v0.2: XPCS from Python*, https://doi.org/10.5281/zenodo.5520626.

[bb38] Perakis, F., Amann-Winkel, K., Lehmkühler, F., Sprung, M., Mariedahl, D., Sellberg, J. A., Pathak, H., *sp*äh, A., Cavalca, F., Schlesinger, D., Ricci, A., Jain, A., Massani, B., Aubree, F., Benmore, C. J., Loerting, T., Grübel, G., Pettersson, L. G. M. & Nilsson, A. (2017). *Proc. Natl Acad. Sci. USA*, **114**, 8193–8198. 10.1073/pnas.1705303114 PMC5547632 28652327

[bb39] Raimondi, P., Carmignani, N., Carver, L., Chavanne, J., Farvacque, L., Le Bec, G., Martin, D., Liuzzo, S., Perron, T. & White, S. (2021). *Phys. Rev. Accel. Beams*, **24**, 110701.

[bb40] Sandy, A. R., Lurio, L. B., Mochrie, S. G. J., Malik, A., Stephenson, G. B., Pelletier, J. F. & Sutton, M. (1999). *J. Synchrotron Rad.* **6**, 1174–1184.

[bb41] Segrè, P., Meeker, S., Pusey, P. & Poon, W. (1995). *Phys. Rev. Lett.* **75**, 958–961. 10.1103/PhysRevLett.75.958 10060161

[bb42] Shinohara, Y., Yamamoto, N., Kishimoto, H. & Amemiya, Y. (2015). *J. Synchrotron Rad.* **22**, 119–123. 10.1107/S1600577514022954 25537597

[bb43] Sutton, M. (2008). *C. R. Phys.* **9**, 657–667.

[bb44] Tong, P., Goldburg, W. I., Chan, C.-K. & Sirivat, A. (1988). *Phys. Rev. A*, **37**, 2125–2133. 10.1103/physreva.37.2125 9899906

[bb45] Weber, R. & Schweiger, G. (1998). *Appl. Opt.* **37**, 4039–4050. 10.1364/ao.37.004039 18273377

[bb46] Williams, M., Busca, P., Collonge, M., Fajardo, P., Fischer, P., Martin, T., Ritzert, M., Ruat, M. & Schimansky, D. (2022). *J. Phys. Conf. Ser.* **2380**, 012091.

[bb47] Zambon, P. (2021). *Nucl. Instrum. Methods Phys. Res. A*, **994**, 165087.

[bb48] Zéberg-Mikkelsen, C. K., Baylaucq, A., Barrouhou, M. & Boned, C. (2003). *Phys. Chem. Chem. Phys.* **5**, 1547–1551.

[bb49] Zhang, Q., Dufresne, E. M., Narayanan, S., Maj, P., Koziol, A., Szczygiel, R., Grybos, P., Sutton, M. & Sandy, A. R. (2018). *J. Synchrotron Rad.* **25**, 1408–1416. 10.1107/S1600577518009074 30179180

[bb50] Zhang, X., Lou, H., Ruta, B., Chushkin, Y., Zontone, F., Li, S., Xu, D., Liang, T., Zeng, Z., Mao, H. & Zeng, Q. (2023). *Proc. Natl Acad. Sci.* **120**, e2302281120. 10.1073/pnas.2302281120 PMC10268294 37276419

[bb51] Zinn, T., Homs, A., Sharpnack, L., Tinti, G., Fröjdh, E., Douissard, P.-A., Kocsis, M., Möller, J., Chushkin, Y. & Narayanan, T. (2018). *J. Synchrotron Rad.* **25**, 1753–1759. 10.1107/S1600577518013899 PMC6225738 30407186

[bb52] Zinn, T., Narayanan, T., Kottapalli, S. N., Sachs, J., Sottmann, T. & Fischer, P. (2022). *New J. Phys.* **24**, 093007.

[bb53] Zinn, T., Sharpnack, L. & Narayanan, T. (2020). *Phys. Rev. Res.* **2**, 033177.

[bb54] Zinn, T., Sharpnack, L. & Narayanan, T. (2023). *Soft Matter*, **19**, 2311–2318. 10.1039/d2sm01334g 36415911

