# Supplementary material for: Improvement of ultra-small-angle XPCS with the Extremely Brilliant Source
Source: J Synchrotron Radiat. 2024 Jan 1;31(Pt 1):65–76. doi: 10.1107/S1600577523008627 (PMC10833426; doi:10.1107/S1600577523008627)
Supplement: Supplementary file 1 [file s-31-00065-sup1.pdf]

JOURNAL OF SYNCHROTRON RADIATION

Volume 31 (2024)

Supporting information for article:

Improvement of ultra-small-angle XPCS with the Extremely Brilliant Source

William Chèvremont, Thomas Zinn and Theyencheri Narayanan

Improvement of ultrasmall-angle XPCS with the Extremely Brilliant Source : Supporting Information

William Chèvremont,[a] Thomas Zinn[a,b] and Theyencheri Narayanan [a]*

[a]*ESRF - The European Synchrotron, 71 Avenue des Martyrs , 38043 Grenoble, France, and* [b]*present address: Diamond Light Source Ltd, Harwell Science and Innovation Campus, Didcot, Oxfordshire OX11 0DE UK*. *E-mail: narayan@esrf.fr*

1. Beam shape and size

In order to determine the illuminated volume of the sample, the beam size has been measured using a direct beam viewer detector placed in the detector wagon (Narayanan *et al.*, 2022). The direct beam viewer is a high resolution CCD camera optically coupled to a high resolution scintillator that converts X-rays to visible light. Images of the beam have been taken at different detector positions, and extrapolated to the sample position. Inspecting the direct beam before XPCS measurements also allows improving the beam shape, avoiding patches and keeping a Gaussian profile.

(a)

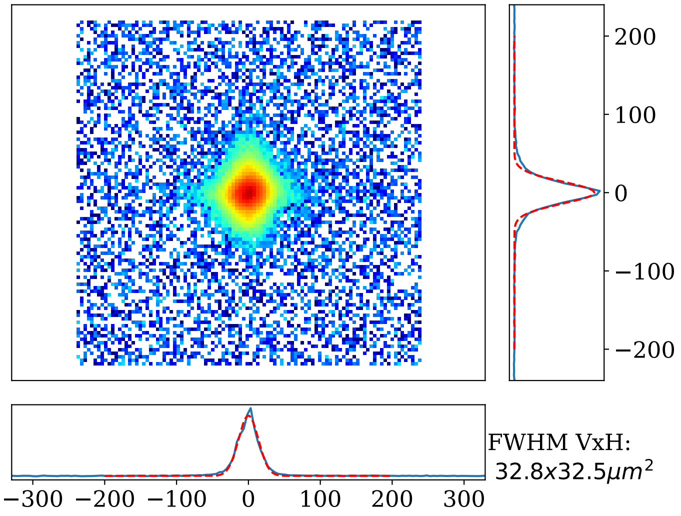

(b)

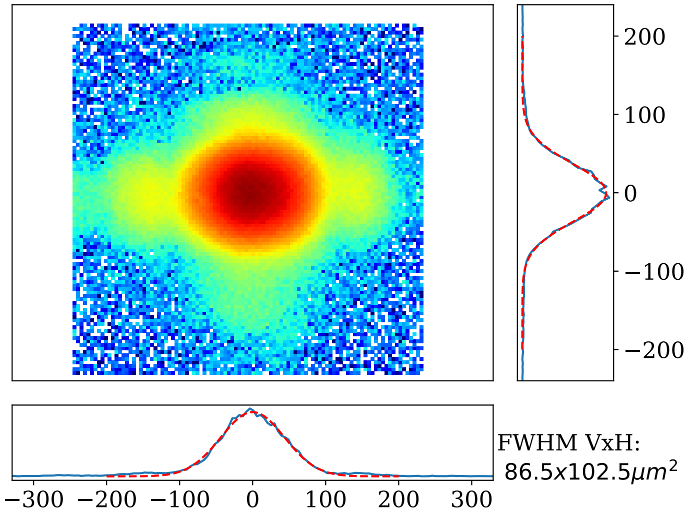

Fig. 1. Direct beam profile at (a) 1 m from the sample position, (b) 31 m from the sample position. The dark image has been subtracted and the colormap is in logarithmic scale. Dashed lines represent fits using Gaussian profiles.

Figure 1 shows the typical profile of the direct beam, in coherence mode for the parameters described in the main manuscript. The profiles have been measured at 1 m and 31 m from the sample position. The profiles from vertical and horizontal slices show a Gaussian shape, and the beam size in terms of full width at half maximum

(FWHM $= 2\sqrt{2\ln(2)}\sigma_B$) can be determined from the fit. In order to determine the beam size at sample position, several beam images are taken close to the sample position, and extrapolated to the sample position. Because of the focusing, the beam size variation is not linear far away from the sample position. The beam size as a function of sample-to-detector distance is described in (Narayanan *et al.*, 2022).

2. Theoretical speckle contrast calculations

The speckle contrast (β) is a sample-independent quantity (Abernathy *et al.*, 1998) defined by the experimental parameters such as the coherence of the incident X-ray beam and the resolution of speckles on the detector. Following the ideas in (Sandy *et al.*, 1999; Lumma *et al.*, 2000; Möller *et al.*, 2019), under certain assumptions that are met in this experimental setup, the contrast can be factorized. y is the axis of the beam, x is the horizontal axis perpendicular to the beam and z the vertical axis. On the $z-$axis $[\beta_z(V_z, \zeta)]$ and on the $xy-$plane $[\beta_{xy}(q, V_x, V_y, \xi, \lambda, \Delta E/E)]$, these factors compare the illuminated volume to the coherence length. $[\beta_q(q, S_y, V_x, \lambda, \Delta E/E)]$ express the contrast of the speckles tilted by the scattering angle (θ) (Hruszkewycz *et al.*, 2012). The last two factors are due to limited angular resolution of the detector, along x and z $[\beta_{res,i}(V_i, L, \lambda, P), i = \{x, z\}]$.

The expressions used for the theoretical speckle contrast calculation are:

$$\beta = \beta_z \beta_{xy} \beta_q \beta_{res,x} \beta_{res,z} \tag{1}$$

$$\beta_z = \left(\frac{\zeta}{V_z}\right)^2 \left[\frac{V_z}{\zeta}\sqrt{\pi}\mathrm{erf}\left(\frac{V_z}{\zeta}\right) + \exp\left(-\frac{V_z^2}{\zeta^2}\right) - 1\right] \tag{2}$$

$$\beta_{xy} = \frac{2}{(V_x V_y)^2} \int_0^{V_y} dy \int_0^{V_x} dx (V_y - y) \exp\left(-\frac{x^2}{\xi^2}\right) (V_x - x) \exp(-|Ax + By|) + \exp(-|Ax - By|) \tag{3}$$

$$A = \frac{\Delta E}{E} q \left(1 - \frac{q^2}{4k^2}\right) \tag{4}$$

$$B = \frac{\Delta E}{E} \frac{q^2}{2k} \tag{5}$$

$$k = \frac{2\pi}{\lambda} \tag{6}$$

$$\beta_q = \left[1 + q^2 \left(\frac{\Delta E}{E}\right)^2 \frac{S_y^2 \cos^2\theta + V_x^2 \sin^2\theta}{4\pi^2}\right]^{\frac{1}{2}}, \theta = \arcsin\left(\frac{q}{2k}\right) \tag{7}$$

$$\beta_{res,i} = \frac{2}{w_i^2} \int_0^{w_x} dv (w_i - v) \frac{\sin^2(v/2)}{(v/2)^2}, w_i = \frac{2\pi P_i}{S_i} = \frac{2\pi P_i V_i}{\lambda L} \tag{8}$$

The integrals are evaluated numerically using the `nquad` function in NumPy suite.

The beamline parameters used to compute the theoretical speckle contrast are provided in Table 1

Table 1. *Main beamline parameters in coherent scattering mode used for the calculation of theoretical speckle contrast values.*

Symbol	Description	ESRF-3rd gen	ESRF-EBS
P	Pixel size	0.075 mm	
R	Source-sample distance	65 m	
$S_i = \lambda L / V_i$	Speckle size		
L	sample-to-detector distance	$1.5 - 31$ m	
V	Illuminated volume along x, y, z	$0.025 \times 1 \times 0.030$ mm^3	
σ_x	Effective source size along x	128 μm	70 μm
σ_z	Effective source size along z	11 μm	9 μm
$\xi = \lambda R / (2\pi\sigma_x)$	Coherence length along x	8.2 μm	14.9 μm
$\zeta = \lambda R / (2\pi\sigma_z)$	Coherence length along z	95 μm	116 μm
λ	Photons wavelength	0.1013 nm	
$\Delta E / E$	Energy dispersion	1.5×10^{-4}	

3. Standard deviation and χ_4

A common quantity to quantify the steady-state in molecular dynamics simulations is $\chi_4(\tau, q)$ that is defined as: (Dasgupta *et al.*, 1991; Glotzer *et al.*, 2000)

$$\chi_4(\tau, q) = \frac{\langle g_{tt}^2(q, \tau, t) \rangle_t - \langle g_{tt}(q, \tau, t) \rangle_t^2}{\beta^2} \tag{9}$$

which is the variance of $g_{tt}(q, \tau, t)$ normalized by the square of experimental speckle contrast. However, β is usually lower than 1 in XPCS, and cannot be determined directly from the data without fitting due to the limited frame rate (the plateau of the autocorrelation function at short lag time might not be accessible). Thus, it is difficult to predict β and thus to measure χ_4 accurately in an online data reduction pipeline (Conrad *et al.*, 2015). Instead, the standard deviation (σ) is computed, which does not make any assumption at the processing time, and it is related to $\chi_4 = \sigma^2/\beta^2$.

4. Speckle contrast reduction at high count rates

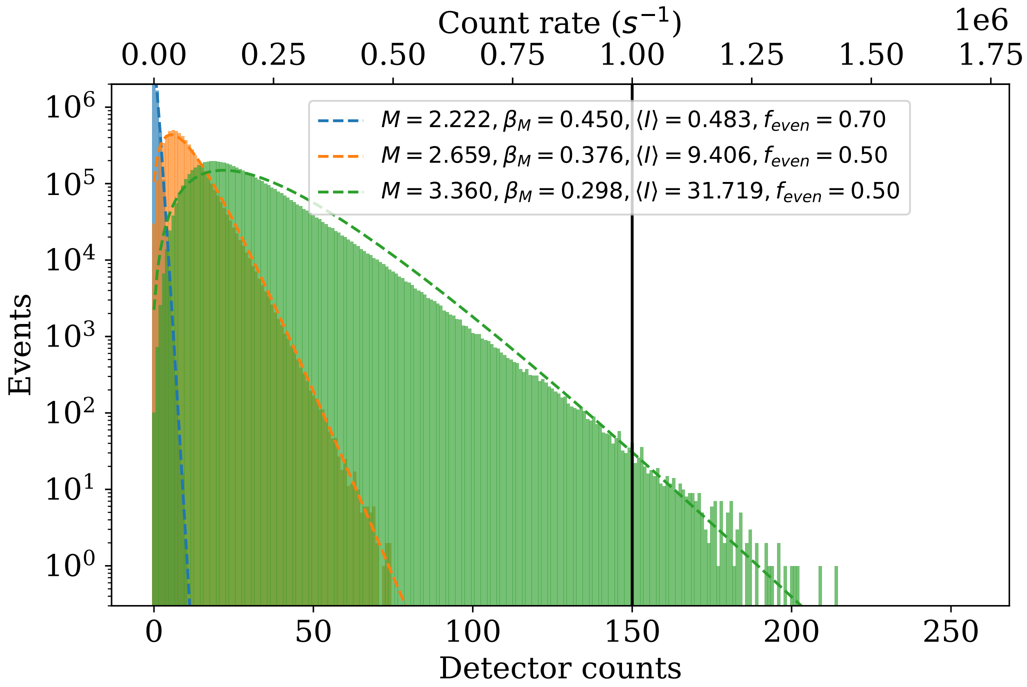

Fig. 2. Histogram of pixel intensities at $q = 1 \times 10^{-2}\,\mathrm{nm}^{-1}$ for the same acquisition as in Fig. 8.

Figure 2 shows the pixel intensity distribution at $q = 1 \times 10^{-2}\,\mathrm{nm}^{-1}$ for the same acquisition as the one presented in Fig. 8 of the main text. At this q, the shape of the distribution for the orange is a Poisson-Gamma function. No crenelation pattern is visible and the relative count of even numbers is much closer to 50%. Compared to Fig. 8(a) in the main text, the contrast recovered the expected value at this q.

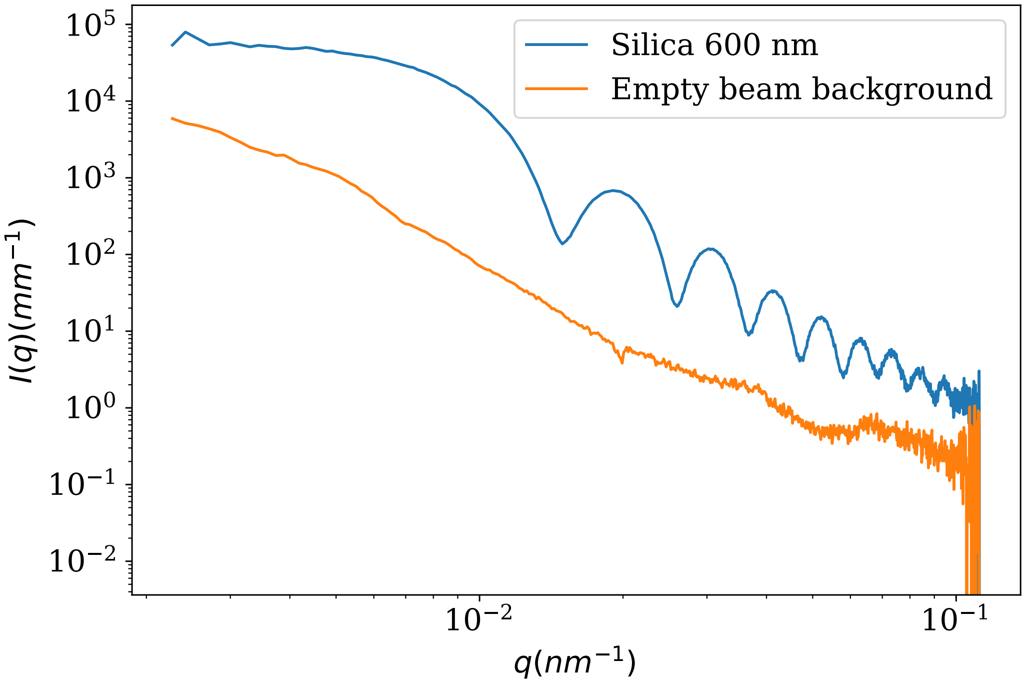

Fig. 3. Unsubtracted SAXS profile from the dilute suspension of 600 nm sized silica particles and the corresponding background.

Figure 3 shows the unsubstracted SAXS profile from the silica colloidal suspension used to measure the impact of high count rate on the intensity statistics and apparent speckle contrast. Even if the minimum of the form factor is about one decade above the background, this still leads to a contrast reduction as shown in the main text. This reduction can be expressed in terms of the relative intensity of the background to the sample scattered intensity.

5. Rheo-XPCS

For the shear cessation experiment, the geometry used was composed of two concentric quartz capillaries, and the X-ray beam traversed just below the rotor. Figure 4(left) shows a scheme of the setup used in the experiment and (right) displays a photo of

the capillary Couette cell mounted on the beamline. This cell has been described in (Narayanan *et al.*, 2020).

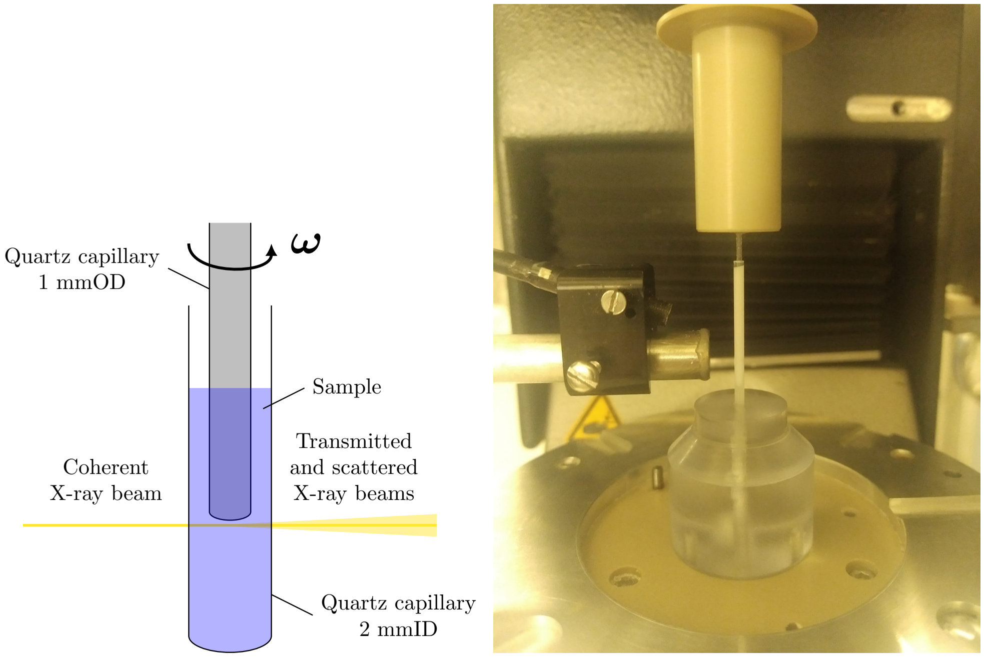

Fig. 4. (left) Scheme of the capillary shear cell used in the shear cessation experiment. (right) Photo of the shear cell on the beamline.

References

Abernathy, D. L., Grübel, G., Brauer, S., McNulty, I., Stephenson, G. B., Mochrie, S. G. J., Sandy, A. R., Mulders, N. & Sutton, M. (1998). *Journal of Synchrotron Radiation*, **5**(1), 37–47.

Conrad, H., Lehmkühler, F., Fischer, B., Westermeier, F., Schroer, M. A., Chushkin, Y., Gutt, C., Sprung, M. & Grübel, G. (2015). *Physical Review E*, **91**(4), 042309.

Dasgupta, C., Indrani, A., Ramaswamy, S. & Phani, M. (1991). *Europhysics Letters*, **15**(3), 307.

Glotzer, S. C., Novikov, V. N. & Schrøder, T. B. (2000). *The Journal of Chemical Physics*, **112**(2), 509–512.

Hruszkewycz, S. O., Sutton, M., Fuoss, P. H., Adams, B., Rosenkranz, S., Ludwig, K. F., Roseker, W., Fritz, D., Cammarata, M., Zhu, D., Lee, S., Lemke, H., Gutt, C., Robert, A., Grübel, G. & Stephenson, G. B. (2012). *Physical Review Letters*, **109**(18), 185502.

Lumma, D., Lurio, L. B., Mochrie, S. G. J. & Sutton, M. (2000). *Review of Scientific Instruments*, **71**(9), 3274–3289.

Möller, J., Sprung, M., Madsen, A. & Gutt, C. (2019). *IUCrJ*, **6**(5), 794–803.

Narayanan, T., Dattani, R., Möller, J. & Kwaśniewski, P. (2020). *Review of Scientific Instruments*, **91**(8), 085102.

Narayanan, T., Sztucki, M., Zinn, T., Kieffer, J., Homs-Puron, A., Gorini, J., Van Vaerenbergh, P. & Boesecke, P. (2022). *Journal of Applied Crystallography*, **55**(1), 98–111.

Sandy, A. R., Lurio, L. B., Mochrie, S. G. J., Malik, A., Stephenson, G. B., Pelletier, J. F. & Sutton, M. (1999). *Journal of Synchrotron Radiation*, **6**(6), 1174–1184.
